# Neuroleptic Malignant Syndrome Status Post Anoxic Brain Injury: A Case Presentation of Heightened Susceptibility in the Brain Injury Population

**DOI:** 10.7759/cureus.35740

**Published:** 2023-03-03

**Authors:** Brianna Cocuzzo, Kristy A Fisher, Clara L Alvarez Villalba

**Affiliations:** 1 Pediatrics, Westchester Medical Center, Valhalla, USA; 2 Psychiatry, Aventura Hospital and Medical Center, Aventura, USA

**Keywords:** acute brain injury, traumatic brain injury, antipsychotics, haloperidol, neuroleptic malignant syndrome (nms), anoxic brain injury

## Abstract

Neuroleptic malignant syndrome (NMS), a potentially life-threatening neurological emergency characterized by muscle rigidity, altered mental status (AMS), autonomic instability, and hyperthermia, is most commonly precipitated by high-potency first-generation antipsychotics due to central dopamine receptor blockade. There is a heightened risk of NMS in animals with ischemic brain injury (IBI) or traumatic brain injury (TBI) due to the resulting death of dopaminergic neurons from injury and the dopamine receptor blockade elicited upon recovery. To the best of our knowledge, this will be the first documented case of a critically ill patient, with a history of prior exposure to antipsychotics, who suffered an anoxic brain injury with subsequent development of NMS after the initiation of haloperidol for the treatment of acute agitation. Further investigation is necessary to expand upon the existing literature suggesting the role of alternative agents, including amantadine, due to its impact on dopaminergic transmission, as well as dopamine and glutamine release. Furthermore, NMS can be difficult to diagnose due to variable clinical presentation and lack of absolute diagnostic criteria, which is further compounded with central nervous system (CNS) injury, where neurological abnormalities and AMS may be attributed to the injury, rather than a medication effect, especially in the early period. This case highlights the significance of prompt recognition with appropriate treatment of NMS in vulnerable and susceptible patients suffering from brain injury.

## Introduction

Neuroleptic malignant syndrome (NMS) is a rare neurological condition characterized by muscle rigidity, autonomic instability, hyperthermia, and mental status changes most commonly secondary to treatment with dopamine-blocking antipsychotics, such as haloperidol [1]. NMS is typically also accompanied by characteristic laboratory abnormalities including elevated creatinine kinase (generally 4× the upper limit), low serum iron, leukocytosis, hyperkalemia, hypomagnesemia, hyponatremia or hypernatremia, metabolic acidosis, elevated blood urea nitrogen (BUN), and elevated lactate dehydrogenase (LDH) [2]. While the pathogenesis of NMS is not fully understood, theories based on clinical manifestations, animal studies, and other supporting data indicate that the development of the condition is strongly influenced by central dopamine receptor blockade. Evidence for this theory comes from several documented medical phenomena including the development of NMS with the use of dopamine-blocking drugs and the development of parkinsonian signs in patients with NMS due to dopamine receptor blockade in the nigrostriatal pathway [2]. Further evidence comes from central dopamine receptor blockade in the hypothalamus as the suspected cause of dysautonomia [2].

Certain populations are at higher risk for the development of delirium, agitation, and psychosis and are therefore more apt to receive treatment via dopaminergic blockade (neuroleptic agents). One such population includes those patients in the acute recovery period following brain injury, both ischemic (IBI) and traumatic (TBI) [3]. Additionally, studies also indicate an increased susceptibility to the development of NMS in this vulnerable population, along with an unexpected response to alleviating medications [4,5]. Furthermore, neuroleptic agents may actually hinder recovery from neurological injury [4]. The proposed pathophysiology encompasses altered dopaminergic signaling and decreased dopaminergic neurotransmission in the striatum and hippocampus of the injured brain [5,6]. However, the mechanism of damage differs between the two distinct types of brain injuries. IBI is via dopamine-mediated damage, while TBI is via diffuse axonal injury, ultimately leading to the loss of neurons, including those within the nigrostriatal pathway [4,6-8].

In the setting of IBI, animal studies demonstrate an initial increase in signal within the striatum, especially when the pathological lesions in the basal ganglia are mild [7]. However, over approximately 72 hours following an ischemic injury, as the pathological lesions in the basal ganglia become more pronounced, this dopaminergic signaling begins to fade [7]. The death of the dopamine-producing striatal neurons ultimately leads to a substantial decrease in dopamine levels with hypoxic-ischemic injuries, which is likely permanent [6]. In TBI, there is a significant disruption of the neuronal networks, including within the nigrostriatal pathway. Of note, even in patients in whom the striatum appears structurally intact, there is decreased dopaminergic signaling in both the striatal dopamine transporter (DAT) and dopamine 2 receptor (DR2). These abnormalities in transmission persist for months [9]. Furthermore, the DR2 are extremely sensitive to hypoxic-ischemic states, which commonly result from TBI or IBI further complicating the body’s response to insult and altering dopaminergic signaling [7]. Thus, we propose this loss of dopaminergic neurons as the responsible mechanism by which brain injury patients are at increased risk of NMS.

## Case presentation

**Figure 1:**
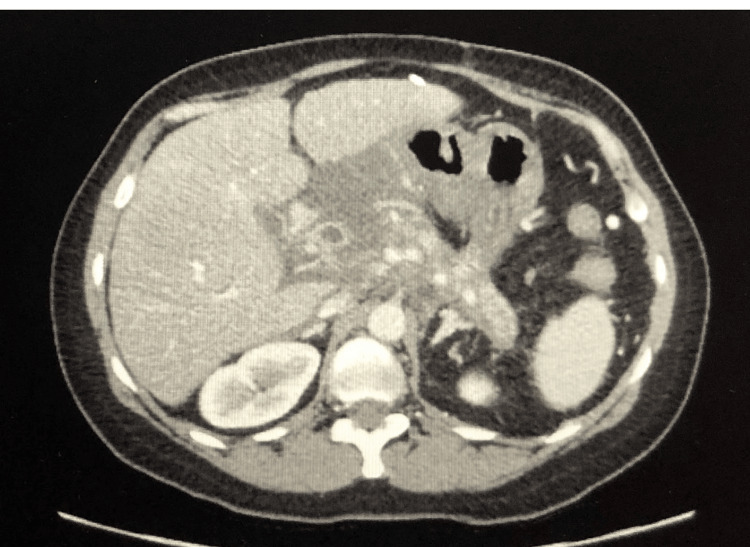
CT of the abdomen confirming the diagnosis of pancreatitis Impression: CT findings compatible with severe acute pancreatitis with extensive pancreatic inflammatory changes and pockets of ill-defined fluid CT: computed tomography

The patient is a 48-year-old female, with a past medical history of hypertension, uncontrolled diabetes mellitus, hypothyroidism, atrial fibrillation, chronic pancreatitis with a pseudocyst, appendicitis, carcinoid tumor, malignant tumor of the right colon with right hemicolectomy, pulmonary embolism, seizure disorder, hydrocephalus with ventriculoperitoneal (VP) shunts status post (s/p) multiple revisions secondary to intraventricular hemorrhage (IVH) while on anticoagulation, gastroesophageal reflux disease, chronic anemia, chronic back pain, bipolar disorder, and panic disorder, who initially presented to the emergency department due to abdominal pain and vomiting for one day. On admission, she was diagnosed with severe acute-on-chronic interstitial pancreatitis secondary to hypertriglyceridemia and admitted for further evaluation and management (Figure 1). Pain alleviation via IV morphine and supportive measures, including IV fluids, was initiated. Of note, upon hospital admission, the patient’s home dose of quetiapine was discontinued, as per the medical team. Metformin was discontinued as well, and an insulin sliding scale was initiated for further management. All other home medications were continued, which included fenofibrate, glyburide, labetalol, and levetiracetam for seizure prophylaxis.

The patient’s hospital course became complicated over the next 19 days when the pancreatic tissue was noted to be infected and necrotic. Empiric antibiotics were initiated. Nasogastric tubal passage was attempted but was unsuccessful due to severe inflammation of the pylorus. Abdominal computed tomography (CT) scan revealed portal and splenic vein thrombosis secondary to venous stasis due to intra-abdominal inflammation. The decision not to anticoagulate was made due to the presence of gastric varices and the increased risk of bleeding. The patient was noted to be febrile with progressive leukocytosis. On day 19, rapid response was called due to encephalopathy, tachycardia, and an oxygen saturation of 80%, which did not improve with a non-rebreather mask or bilevel positive airway pressure (BiPAP) and rapidly required intubation and mechanical ventilation. The patient from there clinically decompensated and suffered acute hypoxic respiratory failure secondary to acute respiratory distress syndrome in the setting of acute pancreatitis and was transferred to the ICU.

**Figure 2:**
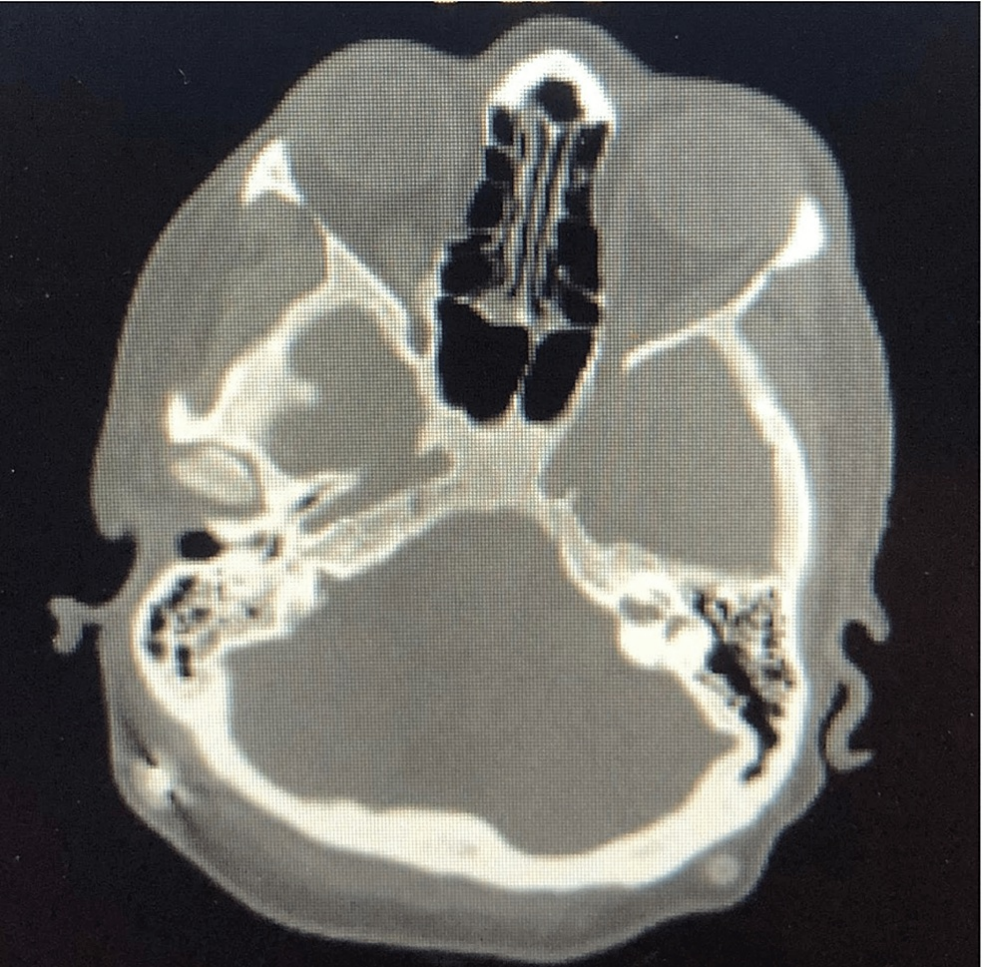
CT of the head confirming chronic subdural hematomas with no acute process A head CT was repeated on 04/06/2020, 04/14/2020, and 05/11/2020 with no reported change in the impression. The above image is from 04/06/2020. Impression: No acute intracranial pathology, no abnormal enhancement, stable bilateral ventriculostomy catheters as described, small chronic bilateral subdural hematomas measuring up to 0.4 cm in depth CT: computed tomography

On day 20, the patient was noted to have left-sided pleural effusion on chest X-ray, which was likely transudative and secondary to pancreatitis. Inhaled ipratropium bromide and albuterol were initiated. Total parenteral nutrition (TPN) was initiated due to the inability to tolerate feeds. On day 21 of her stay, two days after she was intubated, the critical care team weaned the patient off sedation. She made no attempts to verbalize, did not follow commands, and had no purposeful movement. Her neurological examination was limited only to withdrawal from pain and did not buck the endotracheal tube or make attempts to self-extubate. Neurology was consulted for the depressed mental status, and it was determined that the patient suffered an anoxic brain injury due to acute respiratory failure in the context of an already existent extremely poor cerebral reserve. CT of the brain with and without contrast revealed small chronic bilateral subdural hematomas with no acute process (Figure 2). Electroencephalogram (EEG) was also completed, which showed a diffuse cerebral slowing in an encephalopathic pattern. MRI of the brain was ordered by neurology to further characterize this, but unfortunately, it was never completed as the patient was not clinically stable.

On day 22, marked improvement in the patient’s mental status was noted, with opening of the eyes to verbal command and both spontaneous and purposeful movement demonstrated. The patient self-extubated overnight into day 23 and remained extubated. Further improvement in mental status was noted, with the patient’s ability to respond “yes” to pain but inability to specify the exact locale or provide details on severity and type. Unfortunately, this was the best mental status the patient ever regained, and per neurology, her prognosis remains guarded due to continued hypoxic encephalopathy. Over days 23-33, the primary team noted increasing agitation with attempts to remove oxygen lines, as well as attempts to get out of bed. Haloperidol IV pro re nata (PRN) was initiated by the primary medical team, with a total of four doses of 2-3 mg each administered over the course of days 23-24 of hospitalization; haloperidol was then discontinued by the psychiatry team due to concern about cardiac comorbidities. At this time, psychiatry started Depakote (250 mg, three times a day), restarted the home dose of Seroquel (200 mg, at bedtime), and continued Ativan (2 mg, IV Q6 PRN, which she used frequently). On day 33, the psychiatry consultation liaison service was reconsulted for further evaluation of unmanageable agitation over a few days.

**Figure 3:**
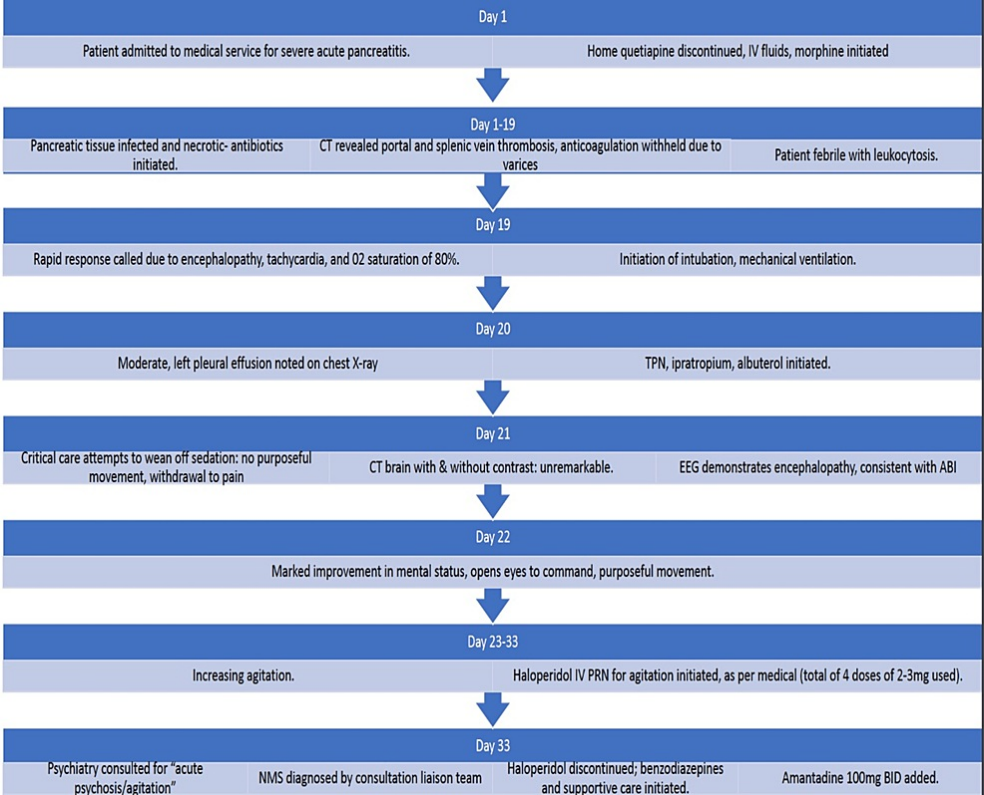
Key points in the patient’s hospital course IV: intravenous, CT: computed tomography, TPN: total parenteral nutrition, EEG: electroencephalogram, ABI: acquired brain injury, PRN: pro re nata, NMS: neuroleptic malignant syndrome, BID: bis in die

The patient’s hospital course from admission to day 33 is shown in graphical form in Figure 3.

Upon initial psychiatric evaluation, the patient was noted to be in acute distress, with altered mental status (AMS)/delirium and agitation, as well as exhibiting signs of catatonia, mutism, rigidity, and dystonia. The patient was noted to be hemodynamically unstable: hyperthermic, tachycardic, and tachypneic, with labile hypertension. Immediate recognition and diagnosis of neuroleptic malignant syndrome (NMS) were established with the support of the initial clinical presentation, physical examination, and indicative laboratory values (Table 1). Furthermore, the nursing staff confirmed a progression of symptomatology typical of NMS, with mental status changes (including both mutism and catatonia) initially, followed by rigidity, then hyperthermia and autonomic dysfunction. Immediately upon recognition of this symptomatology, laboratory results were reviewed and requested to confirm the diagnosis of neuroleptic malignant syndrome.

**Table 1:** The patient’s laboratory values as well as the normal ranges for these laboratory values We postulate that creatinine kinase was not elevated in this patient as expected due to severe muscle wasting.

Laboratory parameter	Patient’s value	Reference range
Serum iron (ug/dL)	14 (low)	50-170
White blood cell count (cells/mm^3^)	26.6 (high)	4.5-11
Lactate dehydrogenase (U/L)	271 (high)	45-90
Blood urea nitrogen (mg/dL)	23 (high)	7-18
Creatinine kinase (U/L)	49 (within normal limits)	10-70

Malignant hyperthermia was ruled out due to the lack of typical causative agents (volatile anesthetic agents and depolarizing muscle relaxants). Serotonin syndrome (SS) was ruled out as the patient was not exposed to agents classically exposed to serotonin syndrome (although cases have rarely been reported with Depakote and Seroquel), as well as the absence of hyperreflexia and myoclonus [2]. The patient’s laboratory findings including leukocytosis, elevated LDH, elevated BUN, and low serum iron lend themselves toward NMS or malignant catatonia (MC). However, these laboratory findings are nonspecific and could also be a feature of the patient’s general medical condition. Therefore, the clinical progression of symptomatology also strongly contributed to making the diagnosis of NMS [2]. Our differential was then narrowed to just NMS and MC; this was the most challenging diagnosis to make as there is a high degree of overlap between them and they are clinically indistinguishable in over 20% of cases [10]. Due to the temporal association of the symptoms to the initiation of haloperidol, the increased risk of NMS in patients with brain injuries, and the lack of prior history of catatonia in this patient or other catatonic features of her presentation, we determined that the more likely diagnosis was NMS. This was confirmed by the patient’s response to the withdrawal of all antipsychotics and initiation of amantadine and Ativan. MC, on the other hand, would not be temporally associated with neuroleptic drugs nor would it respond rapidly to amantadine or low-dose Ativan [10,11]. MC generally does not respond to Ativan as other catatonia does and usually requires electroconvulsive therapy (ECT) for emergent treatment, which this patient did not require [11].

At this time, haloperidol had already been discontinued, but all other antipsychotics were withdrawn, and supportive measures were initiated. Amantadine 100 mg twice daily and Ativan IV 0.5 mg twice daily were also added to the regimen. The patient demonstrated marked improvement regarding NMS presentation over the next two days. However, the hospital course was complicated and prolonged due to the presenting medical condition. The patient ultimately passed away due to medical complications associated with the initial diagnosis of severe acute interstitial pancreatitis.

## Discussion

This case presentation highlights the importance of recognizing and treating NMS, particularly in vulnerable patient populations, such as those with TBI and IBI. We demonstrate a case where a patient with a brain injury was treated with dopaminergic blockade (haloperidol) for the management of agitation. The psychiatry consultation liaison team recognized the symptomatology of NMS, discontinued haloperidol, and initiated medical management. The continued use of haloperidol for agitation management would only have exacerbated the current condition due to decreased dopaminergic signaling in the injured brain, thus causing increased susceptibility to the development of NMS.

Delirium, agitation, and acute psychosis are common manifestations in the acute period following all forms of acquired brain injury, including TBI and IBI [12]. Additionally, this patient population is also at a higher risk for developing psychosis years after injury [3,12]. Among patients hospitalized with acquired brain injuries, many present with acute-onset delirium, especially in the time period of regaining consciousness following an initial comatose state [3,12]. Patients with acute agitation following a brain injury often exhibit restlessness, confusion, disorientation, hallucinations, agitation, and delusions [3,12]. Therefore, the proper treatment of agitation in brain injury patients is crucial not only for optimal management but also for preventing further complications, such as the development of NMS. Currently, a standardized approach to agitation management in such populations does not exist. Furthermore, research suggests that patients with acquired brain injury may not respond as expected to commonly used medications (dopaminergic blocking agents), with exhibitions of either failed response or paradoxically increased agitation to medical management [12].

A systematic review conducted in 2018 demonstrated level 3 evidence that haloperidol may not be effective in managing acute agitation in patients with acquired brain injuries. However, this systematic review also demonstrated level 1a evidence for the use of amantadine in patients with traumatic brain injury [12]. Amantadine indirectly increases dopamine release and prevents the reuptake of dopamine, hence its use in the treatment of Parkinson’s disease [12]. Amantadine also downregulates glutamate, which further contributes to its ability to alter mood and psychosis, including in those patients with neurological dysfunction. While some adverse effects such as rigidity, depression, lethargy, seizures, and ataxia have been reported with amantadine, these symptoms were generally attributed to toxicity, with reports only at doses higher than 200 mg [12]. Further studies are needed to elucidate the dosing of amantadine to manage agitation and aggression in patients after acquired brain injury [12].

This systematic review also provides a class 1b recommendation for the treatment of agitation in acquired brain injuries with beta-blockers [12]. Taken together, evidence from two randomized controlled studies demonstrates that pindolol reduces the number of episodes of agitation in patients with acquired brain injury and propranolol does not reduce the number of episodes of agitation but does reduce the level of the severity of reported episodes of agitation [12]. Further studies are needed to clarify the role of beta-blockers in treating acute agitation following acquired brain injury and determine optimal dosing, but they show promise.

Further consideration is aimed at the proper recognition of NMS due to its ability to mimic other medical conditions, particularly with brain injury patients, where typical manifestations may already go unrecognized [5]. For example, there is a documented report of a 16-year-old male in Alabama who suffered a TBI after a motorcycle accident resulting in a right intraventricular bleed, pontine hemorrhage, left occipital contusion, severe midbrain contusion, and diffuse axonal injury resulting in cerebral edema [5] who was treated with IV haloperidol for agitation and initially believed to have septic shock due to leukocytosis, elevated temperature, and positive sputum culture despite presenting with several features of NMS, including profuse sweating, decerebrate posturing, rigidity with 5/5 strength, poor cognition, dystonic movement of the extremities, increased flexor tone in all four extremities and the hips, increased deep tendon reflexes, bilateral ankle clonus, upgoing Babinski reflexes bilaterally, elevated BUN, elevated lactate dehydrogenase, elevated creatinine kinase, and elevated aspartate transferase (AST) [5]. The patient’s status worsened with the initial treatment of presumed sepsis with vancomycin until the diagnosis of NMS was made, and haloperidol was discontinued. Amantadine, sertraline, and propranolol were initiated. The patient’s fever subsided within 24 hours, and marked clinical improvement was noted. Upon follow-up, the patient was found to be independent with activities of daily living (ADLs) and ambulation and had a successful return to school [5].

NMS can also mimic serotonin syndrome (SS). SS occurs when patients receive multiple drugs that bind serotonin receptors and act as agonists, overloading the receptors and substantially increasing serotonergic neurotransmission [13]. These medications are used in many patients with psychiatric disorders and are frequently co-prescribed with medications that modulate dopamine receptors, increasing the challenge but also underscoring the importance of differentiating these two conditions. There are several sets of diagnostic criteria for SS. To make a diagnosis of SS, patients need at least one feature of the disease, including spontaneous clonus, inducible clonus with agitation or diaphoresis, ocular clonus with agitation or diaphoresis, tremor and hyperreflexia, hypertonia, temperature of >100.4°F, and ocular or inducible clonus [13]. Patients with SS generally also have gastrointestinal (GI) symptoms such as nausea, vomiting, and diarrhea, which are not generally present in neuroleptic malignant syndrome [13]. A diagnosis of NMS, on the other hand, requires elevated temperature and severe muscle rigidity, as well as two or more symptoms, including diaphoresis, dysphagia, tremor, incontinence, changes in the level of consciousness, mutism, tachycardia, labile blood pressure, leukocytosis, and evidence of muscle injury (elevated CK) [2].

NMS and malignant catatonia (MC) can also appear similar clinically, but the progression of the diseases can be different as MC often occurs in patients with a prior history of catatonia and is not generally associated with the initiation of neuroleptic medication. Furthermore, the timeline of symptomatology is different. For most patients with NMS, symptoms of the condition generally manifest within 24-36 hours of the initiation of neuroleptic medications, although the presentation can be delayed. Patients with NMS present initially with mental status changes including both mutism and catatonia, followed by rigidity, then hyperthermia and autonomic dysfunction, exactly as our patient did [10].

Although fever, tachycardia, and altered mental status are present in both neuroleptic malignant syndrome and MC, lead-pipe rigidity, which was present in this patient, strongly favors a diagnosis of NMS. MC, on the other hand, is generally diagnosed when a catatonic patient develops signs of autonomic instability such as fever, tachycardia, diaphoresis, and labile hypertension. Because these patients are catatonic, they generally have waxy rigidity, rather than lead-pipe rigidity. NMS and MC however are similar and, therefore, generally cannot be differentiated on clinical features alone. Consideration of temporal association with medications and response to treatment were the guiding force for the final determination of the diagnosis [14]. The patient was only given 0.5 mg of lorazepam twice a day; this is significantly lower than the dose generally used for catatonic patients, and lorazepam is generally not very effective in malignant catatonia, even at the doses used for retarded or excited catatonia [11]. ECT is generally required for MC; if the diagnosis was MC, the patient would not have recovered so rapidly without the initiation of ECT [11]. Taken together, these findings make it significantly more likely that the patient was having a response to the discontinuation of haloperidol and other antipsychotics and the initiation of amantadine than the initiation of low-dose lorazepam. While there was some improvement with stopping haloperidol, the greatest improvement occurred almost immediately with the initiation of lorazepam and amantadine, suggesting NMS as the correct diagnosis.

## Conclusions

We presented a case of NMS in a non-antipsychotic naive patient after enduring an anoxic brain injury. The postulation of the underlying developmental pathophysiology is a suspected susceptibility to the antipsychotic-induced dopamine blockade due to the reduction of dopaminergic signaling in an injured brain. Although acute agitation is commonly managed with antipsychotics, such as haloperidol, current clinical guidelines and effective managerial options in the management of agitation in the setting of brain injury cease to exist. Further investigation is necessary to expand upon the existing literature suggesting the role of other agents, including amantadine, due to its impact on dopaminergic transmission and both dopamine and glutamine release. Furthermore, NMS is not always recognized immediately, particularly in this vulnerable patient population. This case presentation highlights the significance of prompt recognition with appropriate treatment of NMS in patients suffering from brain injury.
